# Endotracheal intubation in intensive care unit: a prospective study of clinical practice and adverse events

**DOI:** 10.1186/2197-425X-3-S1-A940

**Published:** 2015-10-01

**Authors:** SK Pattnaik, J Nayak, S Choudhary, JP Pati

**Affiliations:** Apollo Hospitals, Bhubaneswar, India

## Introduction

Intubation in critical care unit patients is a difficult proposition due to multiple limiting factors like full stomach, hemodynamic instability,emergency circumstances, difficult positioning, all leading to increased adverse events during intubation.

## Objectives

To describe the practice of endotracheal intubation in the Intensive care unit of a tertiary care teaching hospital, with particular emphasis on the indication, medications used, time of day, staff seniority, number of attempts required and any adverse events.

## Methods

This was a prospective observational study of intubation practice over a 6 months period from 1st October 2014 to 31st march 2015.

## Results

Out of 250 intubations performed with in the time frame, acute respiratory failure (54.5%) was the most common indication followed by trauma. 56.25% intubations done in odd hours (8AM-8PM) having 63.45% success rate on first attempt.

Consultants made the successful first attempt at intubation in 84.5% (95% CI 794.0-88.5), whereas registrars or senior Resident Medical Officers made the first attempt at intubation in 66.6% (95% CI 60.9-71.3).

Complications occurred in 20.10% cases: desaturation, 7.2%; hypotension, 6.5%; aspiration, 3.8%; esophageal intubation, 1.3%; dental injury, 1.2%; and pneumothorax, 0.1%.

Fentanyl plus midazolam combination (70%) was the most common drugs used for intubation.

A bougie was used in 30.9% (95% CI 25.8-36.5) of first attempts, whereas a stylet in 37.5% (95% CI 32.1-43.3).

## Conclusions

Intubation in critical care unit patients is a risky procedure which needs a standard operating guidelines to decrease the adverse events.

